# Eclectic Department

**Published:** 1853-12

**Authors:** 


Dr. Robert I. Graves recommends in the Dublin Quarterly, August, 1852, a saturated solution of gutta percha in chloroform, in cutaneous diseases. Another surrogate of collodion is recommended by Dr. Wellez, in Beecher's Repert., vol. vi., No. 3, viz: A solution of shellac in hot alcohol. He assures it to be more adhesive than collodion, to contract strongly while drying. It is impermeable by air, water, or oil, does not irritate the skin, and is cheaper than collodion.

